# Combined chlorhexidine-sodiumfluoride mouthrinse for orthodontic patients: Clinical and microbiological study

**DOI:** 10.4317/jced.51979

**Published:** 2015-12-01

**Authors:** Mahboobe Dehghani, Mostafa Abtahi, Hamed Sadeghian, Hooman Shafaee, Behrad Tanbakuchi

**Affiliations:** 1 Assistant professor of orthodontics, Dental research center, School of Dentistry, Mashhad University of Medical Sciences, Mashhad, Iran; 2 Associate professor of orthodontics, Dental research center, School of Dentistry, Mashhad University of Medical Sciences, Mashhad, Iran; 3 Pathologist, Department of general Pathology, Faculty of Medicine, Mashhad University of Medical Sciences, Mashhad, Iran; 4 Assistant professor of orthodontics, Department of orthodontics, School of Dentistry, Tehran University of Medical Sciences, Tehran, Iran

## Abstract

**Background:**

Orthodontic appliances impede good dental plaque control by brushing. Antimicrobial mouth rinses were suggested to improve this performance. We therefore aimed to investigate the effects of combined mouthrinse containing chlorhexidine (CHX) and sodium fluoride (NaF) on clinical oral hygiene parameters,and plaque bacterial level.

**Material and Methods:**

In this double-blind clinical study, 60 fixed orthodontic patients aged 14-25 years were randomly assigned to one of four mouthrinses groups: 1- combined CHX /NaF 2- CHX 0.06% 3- NaF0.05% 4-placebo. Following baseline examination patients were instructed to use the assigned mouthrinse twice daily for 21 days. Bleeding index (BI), modified gingival index (MGI) and plaque index (PI) were determined at the baselineand after three weeks of rinsing. Samples from supragingival plaque were obtained for the assessment of total bacterial, *Streptococcus mutans* and *Lactobacilli* colony counts. Data were analyzed by Wilcoxon, Kruskal-Wallis, and Mann-Whitney tests.

**Results:**

Clinical parameters; All three active mouth rinses induced significant improvements of BI, MGI, and PI (*P*<0.05). Results of CHX/NaF were slightly, but not significantly, better than CHX. CHX/NaF and CHX induced significantly more changes than NaF and placebo. Microbiological measurements; Except placebo, other mouthrinses reduced total bacterial, *Streptococcus mutans*, and *Lactobacilli* counts significantly (*P*<0.05). CHX/NaF acted against *Lactobacilli* significantly more than others.

**Conclusions:**

Adding CHX0.06%/NaF0.05% combined mouth rinse to daily oral hygiene regimen of orthodontic patients significantly improved oral hygiene status. Effect of this combined mouth rinse on dental plaque *Lactobacilli* was remarkable. However, large controlled trials could provide more definitive evidence.

** Key words:**Mouthrinse, fluoride, chlorhexidine, plaque.

## Introduction

Dental plaque is the major etiologic factor in the development of dental caries and gingivitis which typically accumulated during the orthodontics (1,2). Orthodontic metallic attachments cause alterations in the oral microflora by PH decreasing, bacteria affinity to the metallic surfaces due to the electrostatic reactions, and creating new plaque retentive areas which in turn predisposes to increased microbes carriage (3). Generally orthodontic patients are unable to maintain adequate oral hygiene by mechanical means alone due to the failure of plaque removal from difficult to access areas that are hindered by orthodontic attachments (4). A common strategy is to add a chemotherapeutic agent such as antimicrobial mouthrinses into mechanical oral hygiene regimen (2,5,6). Mechanical means remove bulk of plaque, while remaining plaque may be inactivated by antimicrobial mouthrinses. They act against plaque by either of preventing bacteria adhesion, disturbing bacterial vitality or disrupting existing plaque (7,8).

Streptococcus mutans and *Lactobacilli* are known as most closely bacteria associated with dental caries (9-11). Shortly after bonding orthodontic attachments, level of these bacteria in the oral cavity elevates due to plaque accumulation (12,13). Reduction of these cariogenic bacteria is an important step to prevent caries (11). It has been shown that chlorhexidine (CHX) and fluoride mouthrinses have activity against oral pathogens.

CHX is the most popular antimicrobial mouthrinses. There is strong evidence to support antiplaque and antigingivitis effects of CHX (5,14). It has antimicrobial effects against the periodontal and cariogenic pathogens *streptococcus mutans* (*S.mutans*) and *Lactobacilli* (6,11,15). Previous studies have shown a significant reduce in the amount of plaque and also gingivitis in orthodontic patients who received CHX mouthrinse (4,16).

Caries-preventive and cariostatic effects of fluoride have been extensively accepted (17). Widespread use of fluoride is the most common reason of dental caries decline in western countries in recent years. Fluoride accumulates in dental plaque and decrease amount of plaque and gingivitis. Fluoride has antibacterial activity against *S.mutans* (18,19).

Given that both CHX and fluoride have antimicrobial activity and are effective against dental caries and gingivitis, it was hypothesized that a combination formula would provide a strengthening effect. To date limited information is available regarding evaluation combined CHX/fluoride formulation effect. The few available studies assessed mainly its effects on gingival parameters (20,21). Although assess antiplaque and antigingivitis effects of mouthrinses is valuable, it is also of interest to investigate the efficacy on specific plaque bacteria that play a significant role in dental diseases. Considering benefits of two materials, more research is required on the subject.

The objective of the present study was to assess clinically and microbiologically the efficacy of three weeks rinsing with a combination mouthrinse containing both CHX and sodium fluoride, CHX mouth rinse, and sodium fluoride mouth rinse in patients undergoing fixed orthodontic treatment.

## Material and Methods

In this study, 60 orthodontic patients who were under fixed orthodontic treatment in the department of orthodontics of Mashhad University of Medical Sciences in Mashhad, Iran were participated. The research protocol was approved by the Ethics Committee of the University. After explaining the aim and process of the study to the volunteers, informed signed consent was obtained from the parents. The study was a double-blind parallel-group clinical trial.

-Inclusive criteria were:

• In the age range of 14-25 years.

• Willingness to participate in the study

• Mild gingivitis

• Full bonded edgewise treatment with brackets on anterior teeth and premolars and bands on first molars.

-Exclusive criteria were:

• Antibiotic therapy in last month

• Medical problems 

• Pregnancy and lactation

• Smoking

• Moderate or severe gingivitis/priodontitis

• History of hypersensitivity to mouthrinses

• Using any mouthrinse in last month.

All selected subjects had mild gingivitis at the start of study. Two tables of random numbers, one for the male population and one for the female population were used and the subjects were thus randomly assigned to one of four treatment groups (n=15 in each group). The groups and assigned mouthrinses were as follows:

A: combined mouthrinse containing chlorhexidine digluconate 0.06% and sodium fluoride 0.05% 

B: CHX mouthrinse containing chlorhexidine digluconate 0.06%

C: NaF mouthrinse containing sodium fluoride 0.05%

D: placebo mouthrinse

The mouthrinses were dispensed through other staff of the department due to the double-blind design of the study. All of the mouthrinses had similar bottle appearance.

Participants were asked to rinse twice a day in the morning and evening after brushing for three weeks. Patients were instructed to rinse with 15 mL of the solution for 1 min followed by expectoration of the residual mouthrinse and avoid drinking and eating till 30 minutes. To avoid the effect of new variables, subjects were asked to continue their usual daily brushing method during the study period. The subjects refrained from oral hygiene procedures as well as eating and drinking in the morning of the first day (baseline measurements) and 22th day (final measurements).

For clinical parameters, scores including Bleeding Index (BI), Modified Gingival Index (MGI), and Plaque Index (PI) were taken from all participants by the same blinded trained examiner at baseline and 22th day. The examiner was a senior resident of orthodontics. Before the study the examiner was calibrated in the use of periodontal indices by an experienced periodontist. The measurements were recorded from central incisors, canines and second premolars of four quadrants. BI was scored as Saxton and van der Ouderaa (22) upon probing the buccal sulcus of mentioned teeth as described: 0=absence of bleeding after 30 seconds, 1=bleeding after 30 seconds, 2=immediate bleeding.

The MGI was scored on the buccal marginal gingiva of above teeth according Lobene *et al.* definition as following degrees of inflammation: 0=No, 1=mild (either marginal or papillary gingival unit), 2=mild (entire marginal and papillary gingival unit), 3=moderate, 4=severe (23).

The PI was scored on buccal surface according to the Turesky modification of the Quigley-Hein PI as follows: 0=no plaque, 1=discontinuous band of plaque at the gingival margin, 2=up to 1 mm continuous band of plaque at the gingival margin, 3=band of plaque wider than 1 mm but less than one-third of surface, 4=plaque covering one-third or more of the surface, but less than two-thirds of the surface, 5=plaque covering two-thirds or more of the surface (24).

A mean value of each parameter was calculated in each of pre and post rinse measurements.

For microbial evaluation, supragingival samples were collected cervically from the bracket on the second premolars, in both jaws at baseline and after three weeks. Plaque sampling was performed by using a sterile curette. Samples were transferred separately into vials containing 1mL of TSB (Tripticase Soy Broth) and immediately brought to the Microbiology Laboratory of an academic Hospital (Mashhad, Iran) for further processing. Then, 50µL of the specimen were cultured on Blood Agar and EMB mediums. After 48hrs incubation at 370c colony counts were enumerated. Total bacterial count was determined by visual counting and the latter was multiplied by 20 to express as colony forming units(CFU)/mL. For specific colony counts, *S.mutans* and *Lactobacillus* colonies were morphologically identified and enumerated as described above. The colonies were further confirmed by different microscopic and biochemical tests such as Gram stain, Bile esculin hydrolysis, Bacitracin and Optochin tests.

-Statistical methods:

Statistical analysis was performed using SPSS (version 15) software. The values of bacterial counts (CFU/mL) were transferred into log10 values before statistical analysis. According Kolmogorov-Smirnov Test, data distribution in both clinical and microbial values was nonparametric. In each group, pre- and post-rinsing values were compared by Wilcoxon Signed Rankstest. For each parameter, Kruskal-Wallis test was used for comparison the differences in median ranks among study groups. Pairwise comparison of the differences between groups was performed by nonparametric Mann-Whitney test followed by corrected Bonferroni method. The significance level was set at 5 percent.

## Results

In this study, 60 subjects were participated. There were 27 (45%) males and 33 (55%) females with the mean age of 16.38 ± 1.45 years (age range= 15-22 years).

 Table 1 shows mean and median BI, MGI, and PI values for each of study groups at baseline and end of study protocol. The “difference” indicates substraction of pre- and post-rinsing values. In combination, CHX and NaFmouthrinses groups, all clinical values in median ranks decreased from pre-rinsing to post-rinsing except than PI of NaF group. Comparing the “difference”s by Kruskal-Wallis test demonstrated that there were significant differences among four groups in all three clinical parameters (*P*<0.001).

**Table 1:**
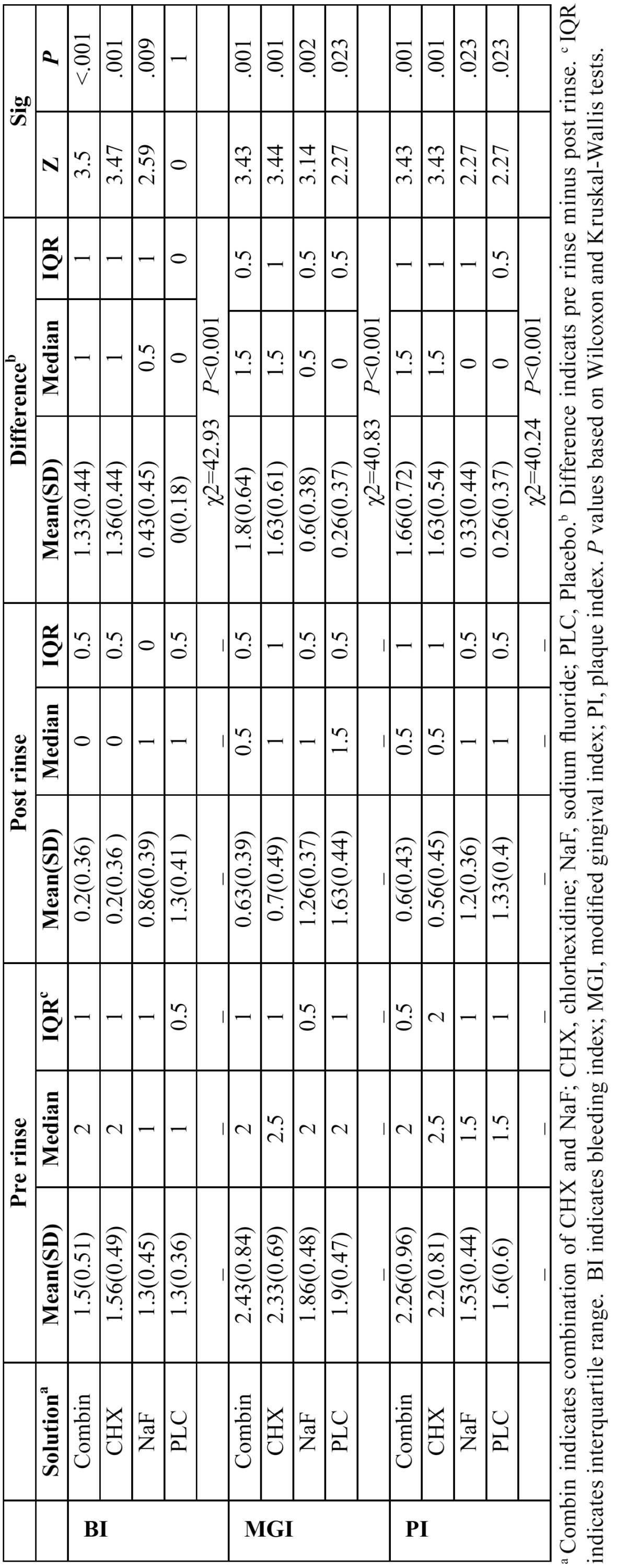
Mean and median BI, MGI, and PI values in different study groups at baseline and 3 weeks.

 Table 2 demonstrates the results of the Mann-Whitney analysis to define where the differences. Clinical parameters in combined mouthrinse group were not significantly different from CHX, while both were significantly different from each of NaF and placebo groups (*P*<0.001).

**Table 2:**
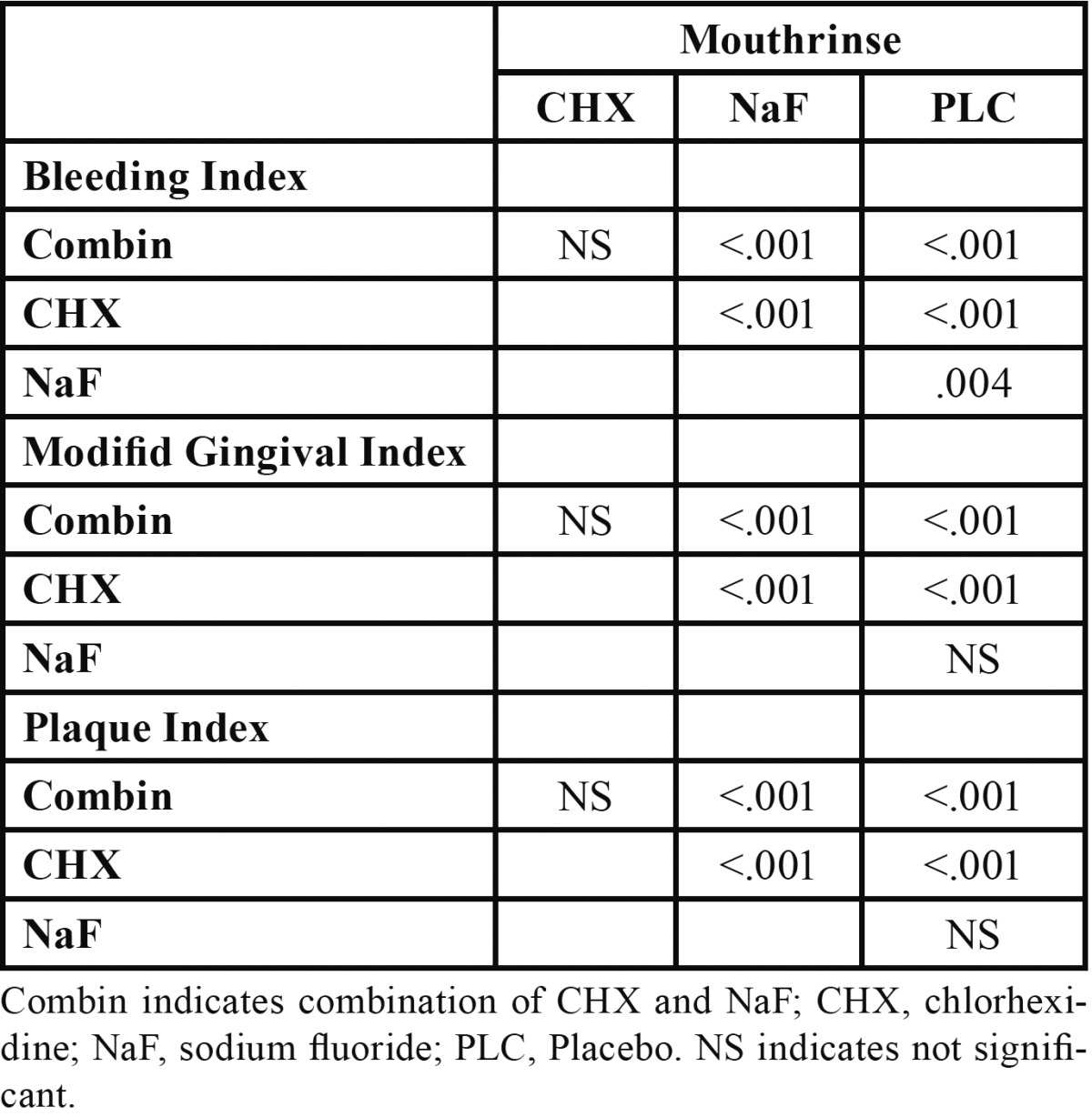
Intergroup comparisons of four mouthrinses based on clinical scores (Mann-Whitney analysis).

Mean and median values of bacterial counts at baseline and end of 3 weeks and the “differences” of pre- and post-rinsing are shown in  Table 3. Statistical analysis showed significant differences among four groups for total bacterial counts, *Streptococcus mutans* counts and *Lactobacilli* counts (*P*<0.001, *P*<0.001, *P*=0.002 respectively).

**Table 3:**
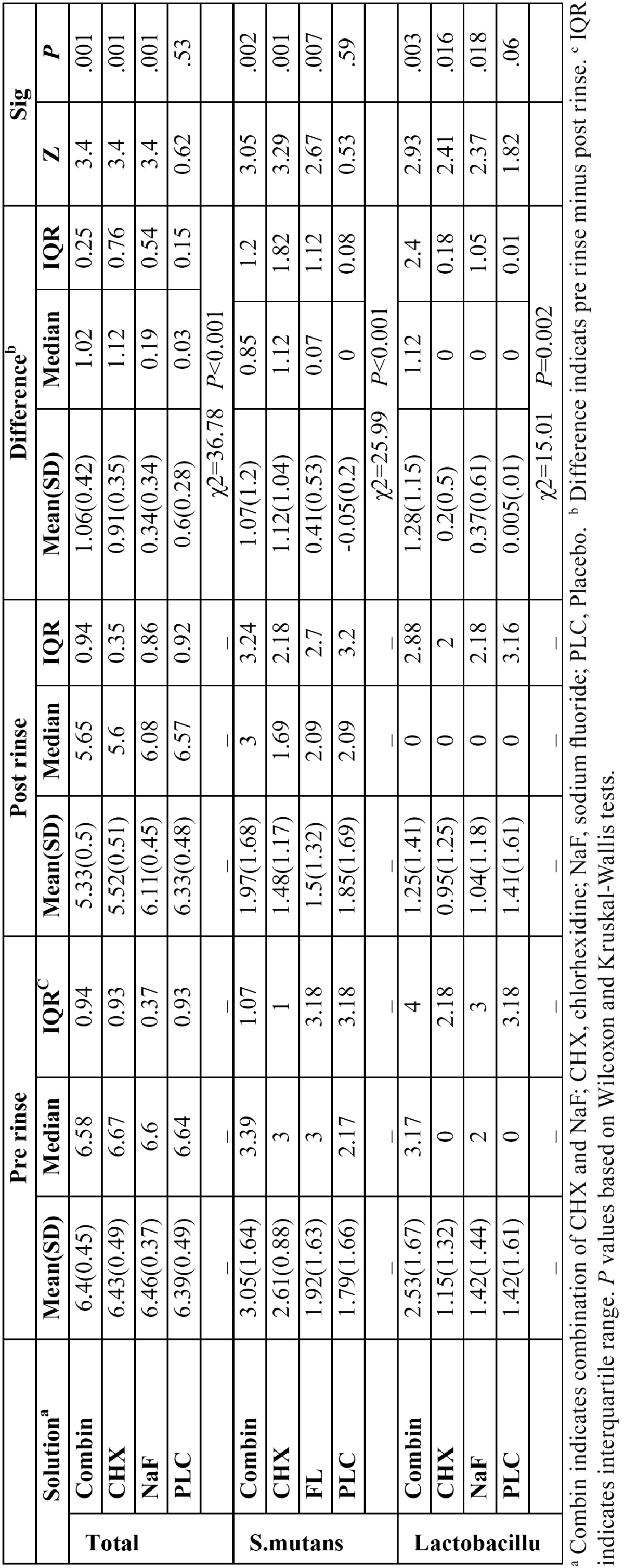
Mean and median CFU/mL (log10) of Total Bactria, Streptococcus mutans, and Lactobacillus in different study groups at baseline and 3 weeks.

Intergroup comparisons of bacterial counts results are demonstrated in  Table 4. All comparisons in total bacterial counts, showed statistically significant differences except the difference between combined mouthrinse group and CHX only. Regarding the *S. mutans* counts; the differences of placebo group with other threes were significant. In *Lactobacilli* counts, changes induced by combined one were significantly more than CHX, NaF, and placebo. Subjects did not experience any adverse effect or dental staining during the study.

**Table 4:**
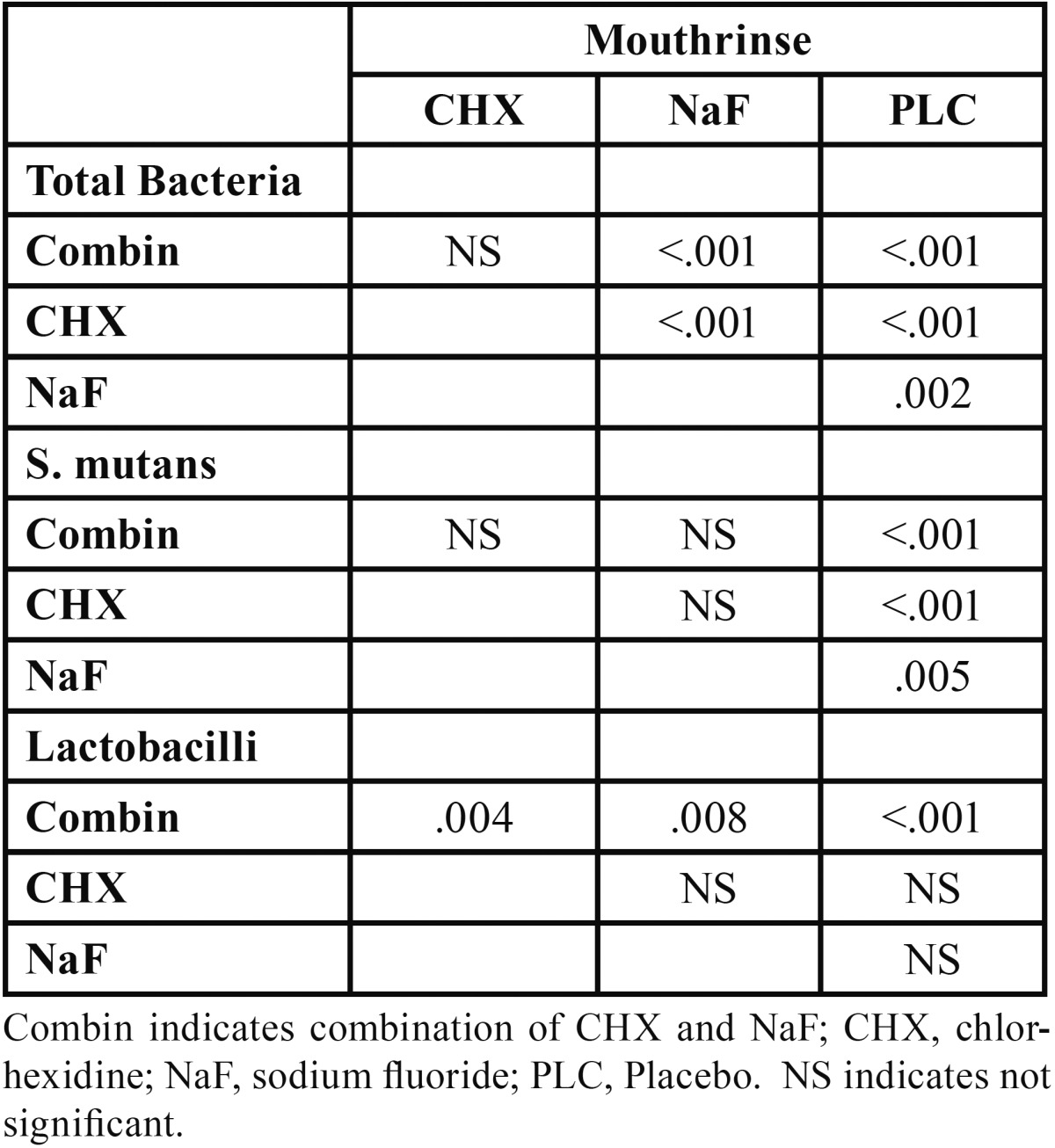
Intergroup comparisons of four mouthrinses based on microbiological parameters (Mann-Whitney analysis).

## Discussion

Evidence shows significant improvement of oral hygiene status when antimicrobial mouthrinses are added to daily oral hygiene regimen and the importance of such an agent is even greater in orthodontic patients (4). Prescription of antimicrobial mouthrinses in such cases, who are struggled to brush and particularly to floss in the presence of brackets and wires, may be significantly beneficial. Moreover, these patients are considerably susceptible to initiation of dental caries and gingivitis. CHX mouthrinse and fluoride mouthrinse were recommended to be used routinely. Combining these two mouthrinses into a one would surely facilitate the usage of the liquid. The present study was therefore designed to evaluate clinical and microbiological effects of CHX/NaF combined mouthrinse, CHX mouthrinse and NaF one when added to oral hygiene regimen of orthodontic patients.

In order to obtain the best possible evidence, double-blind design was chosen. The design was intended to evaluate the effect of mouthrinses, so nothing rather than mouthrinse administration was changed; therefore no oral prophylaxis, no oral hygiene instructions and no new toothbrush/toothpaste were given. On the contrary, in many related reports, the instructions and same brand brushing means were given at the start which might motivate cases to improve their oral hygiene during the study which is an additional variable (2,4,13). In such situations, to allocate the results merely to the mouthrinse is questionable.

The study population comprised teenagers and young adult orthodontic patients. Taking into consideration that these patients fear from dental caries during orthodontics and have difficulty in mechanical plaque control, it seems that they are an interesting group for this kind of studies in terms of compliance. In order to observe better the efficacy of mouthrinses, all selected subjects had mild gingivitis at the start of study.

There are few full reports published on the efficacy of combined CHX/NaF mouthrinse. After literature review, few reports were found to study the effect of combined mouthrinse, mainly in clinical periodontal parameters (20,21). So far, this is the first published trial that assessed the clinical and microbiological effects of rinsing by combined CHX0.06%/NaF0.05%. Subsequently, the limited number of published studies on the subject will make the comparison of our results with other groups rather restricted.

According to the clinical part of the study, a general improvement in clinical parameters was observed in active mouthrinses groups. Combined group effects were near to CHX one. It should be mentioned that although statistically CHX/NaF was not superior to CHX, generally the former induced more changes. Both were significantly stronger than NaF; this does not necessarily rule out the therapeutic effects of fluoride, but probably is due to dominant antimicrobial role of CHX. Currently CHX recognized as gold standard mouthrinse (14). Antiplaque and antigingivitis results of CHX are as expected and are in agreement with the previous studies (4,5,16). However, concentration of CHX was 0.06% which is lower than most previous works (4,16,25).

Combining CHX/NaF did not show more clinical benefits than CHX alone in short-term. However, long-term uses may be different. Jayaprakash *et al.* (20) concluded that in a short-term, antiplaque and antigingivitis effects of CHX/NaF were not significantly different from CHX alone, but in a long-term, results of the combined therapy was more satisfactory. Apparently, this indicates more antimicrobial advantages of fluoride in prolonged uses. Fluoride effects may be evaluated better by caries-specific parameters such development of white spot lessions which need more prolonged studies.

According to the microbiological assessment, all three active mouth rinses induced significant improvements. In accordance with the results of previous studies (6,8), present study showed CHX efficiently act against plaque bacteria. CHX antimicrobial effect is well-recognized. CHX prompts changes to cell membrane function and leakage of intracellular constituents (26). Certainly, previous researches mainly showed efficacy of 0.2% and 0.12% concentration of CHX (6,8,13), while current study indicated that lower concentration of CHX(0.06), alone or in combination with NaF0.05%, is also efficient against plaque bacteria such as *S.mutans* and *Lactobacillus*. These findings further support the idea of previous work, indicating that even 0.02% and 0.06% concentration of CHX effectively reduce *S.mutans* (15).

This study clearly demonstrates anticariogenic effects of NaF with regard to bacterial counts. Both *S.mutans* and *Lactobacillus* significantly affected by 0.05% NaF. Effectiveness of NaF against *S.mutans* has been pointed out in the literatures. Del Carmen *et al.* (27) reported an inhibition of *Lactobacillus* by NaF while some others (18,19) stated NaF has no effect on it. In this study, efficiency of NaF against *Lactobacillus* can be explained considering general cariogenic effects of fluoride.

In current study, *Lactobacillus* less than *S.mutans* reacted to CHX which confirm Sari and Birinci findings (13). This corroborates the claim that *Lactobacillus* have a low sensitivity to CHX(12).

Despite the *Lactobacillus* partial resistance to CHX and NaF, combined mouthrinse has dramatic influence on it. Present findings indicate that combined CHX/NaF does not have any more effects on *S. mutans* compared to CHX alone. However, the CHX/NaF formula showed synergistic effect on *Lactobacillus* and reduced its count significantly. Due to of the limited number of published study on effects of combined formula against specific bacteria, these results may not compare with others truly. Giersten and Scheie reported that CHX/NaF mouthrinse reduces lactate formation in plaque bacteria as compared with NaF mouthrinse (28).

Dental staining is one of side effects of CHX, however patients in any of CHX/NaF and CHX groups did not complain of staining. It may be justified by lower concentration of CHX in current study (0.06) compare to usual 0.2% and 0.12% CHX. If lower concentrations are as effective as higher ones, it will be a better option due to the lower side effects (15,25). Further experimental investigations are needed to focus more on this subject.

It may be concluded that observed findings was attributable to the fact that the subjects knew that they are being studied or to the mechanical effect of rinsing alone. Given that there was no improvement in the scores of placebo group, the active mouth rinses effects could not be attributed to Hawthorne effect or to mechanical effect of rinsing alone (29). Nevertheless, in thecurrent study, adding NaF to CHX mouthrinse had not any adverse effect and generally, not all significantly, improved results in comparison with CHX and NaF alone. Furthermore its effect on *Lactobacilli* was remarkable. However, this research was relatively a short clinical study and the findings may be different to the prolonged one. It is recommended that the assessment to be performed in the long-term significance of using combined CHX/NaF mouthrinse in orthodontic patients. Finally, it should be noted that chemical agents such as mouthrinses are not substitutes for through brushing and flossing, but they should be used as adjuncts.

As conclusion, within the limitations of this study, adding Chlorhexidine 0.06%/ sodium fluoride 0.05% combined mouthrinse to daily oral hygiene regimen of fixed orthodontic patients significantly improved oral hygiene status clinically and microbiologically. In comparison with CHX mouthrinse and NaF mouth rinse, the effect of combined one on dental plaque *Lactobacilli* was remarkable with no adverse effect. However, further long-term studies are highly recommended to prove the efficacy of combined CHX 0.06%/NaF 0.05% mouthrinse.
